# A133 PORTAL VENOUS STENTING FOR THE TREATMENT OF NON-CIRRHOTIC PORTAL HYPERTENSION-RELATED GASTRIC ANTRAL VASCULAR ECTASIA: A CASE REPORT

**DOI:** 10.1093/jcag/gwac036.133

**Published:** 2023-03-07

**Authors:** M Deeb, F Almousawi, G Annamalai, C Barragan Leal, S Wolman, F Habal

**Affiliations:** 1 Division of Gastroenterology, Department ofMedicine, University of Toronto; 2 Division of Vascular and Interventional Radiology, University Health Network, Toronto, Canada

## Abstract

**Background:**

Pancreatic and hepatobiliary cancers account for 15-24% of extrahepatic portal hypertension. Treating portal hypertensive bleeding associated with these tumors has been challenging with the delayed effect of chemoradiation and patients with active bleeding being unfit for such therapies.

Portal vein stenting has been described in the literature for the treatment of variceal bleeding in such patients. We present the first report of its use for the successful treatment of refractory gastric vascular ectasia (GAVE)-related bleeding.

**Purpose:**

To review portal vein stenting as a treatment for non-cirrhotic portal hypertension-related GAVE bleeding.

**Method:**

Chart review was performed with the patient's informed consent.

**Result(s):**

A 53-year-old female patient with locally invasive pancreatic cancer presented with one week of melena and presyncope. She had a history of gastroesophageal reflux disease. Her tumor encased the celiac trunk, hepatic and splenic arteries, and abutted the superior mesenteric artery. She had been treated with chemotherapy and radiation.

On examination, she was afebrile, blood pressure was 132/87, pulse 110 beats/min, and oxygen saturation 99% on room air. Her abdomen was soft and non-tender. Her hemoglobin was 63 g/L, which represented a 64-point drop over 4 months, leukocytes 4.5 x10^9^/L, platelets 238 x10^9^/L, INR 1.1 and normal electrolytes and creatitine. Alkaline phosphatase was 399 U/L, aminotransferases normal, albumin 38 g/L and total bilirubin 6 umol/L. She was started on pantoprazole and octreotide. On admission, gastroscopy revealed copious blood in the stomach with no clear source despite gastric lavage; hemospray was applied. Given persistent bleeding, repeat gastroscopy the following day revealed 4 columns of Baveno grade III esophageal varices with no high-risk stigmata, as well as severe portal hypertensive gastropathy and GAVE. Argon plasma coagulation and esophageal banding were performed. Over the next month, she continued to have maroon-colored stools. CT angiogram revealed portal vein occlusion and contrast extravasation within the gastric lumen, in keeping with her known gastric antral bleed, with no evidence of lower gastrointestinal bleeding. She had 5 further gastroscopies where APC and GAVE banding were performed. Despite that, she developed hematemesis. Interventional radiology was consulted and performed transhepatic portal venous recanalization, angioplasty and stenting using a 10mm x 60mm self expandable nitinol stent from the superior mesenteric vein to the main portal vein. After stenting, previously seen collateral veins and cavernous transformation were significantly reduced on angiography. She was started on clopidogrel to maintain stent patency. Two days following stent insertion, she stopped clinically bleeding and her hemoglobin stabilized. She was discharged home one month later.

**Conclusion(s):**

In patients with GAVE-related bleeding refractory to standard therapeutics, portal venous stenting is possibly an effective treatment modality.

**Please acknowledge all funding agencies by checking the applicable boxes below:**

None

**Disclosure of Interest:**

None Declared

